# Enhanced Ovarian Folliclular Development by Metformin Does Not Correlate with Pregnancy Rate: A Randomized Trial

**Published:** 2012-06-19

**Authors:** Zahra Basirat, Mehrdad Kashifard, Masoumeh Golsorkhtabar Amiri

**Affiliations:** 1 Fatemezahra Infertility and Reproductive Health Research Center, Babol University of Medical Science, Babol, Iran; 2 Department of Internal Medicine, Babol University of Medical Science, Babol, Iran

**Keywords:** PCOS, Ovulation Induction, Metformin, Clomiphene Citrate.

## Abstract

**Background:**

Polycystic ovary syndrome (PCOS) is a common, complex endocrine disorder for women of productive age. A high incidence of ovulation failure in women with PCOS is related to insulin resistance. Some studies have assessed the effects of hyperinsulinemia and insulin resistance in relationship with insulin sensitizing agents such as Metformin (Met). These medicines have been suggested new scope for ovulation stimulation enhancement with Clomiphene Citrate (CC) in PCOs women. The aim of this study is to compare the effectiveness of adding Met to CC in women with PCOS.

**Materials and Methods:**

This multicenter, single-blind, randomized controlled trial study was performed on 334 PCOS patients from 2007 to 2009. Patients were randomly divided into two groups and ovulation induction was performed with either CC alone or CC + Met. The treatment was continued for three cycles, then the mature follicle and pregnancy rates were evaluated.

**Results:**

In the CC + Met group, 68% had at least one dominant follicle in the first cycle that was significant (p<0.001), and 31.7% had one in the second cycle. In the CC group 54.5% in the first cycle, 31.7% second cycle, and 6.9% ovulated in the third cycle. The pregnancy rate was 28.7% in CC + Met group and 24.6% in the CC group, with no significant differences between the two groups.

**Conclusion:**

Adding Met to CC is significant for ovulation, but it does not enhance the pregnancy rate (Registration Number: IRCT138904174306N1).

## Introduction

Polycystic ovarian syndrome (PCOS) is the most common cause of infertility, affecting approximately 5%-10% of women of reproductive age (1). It is also considered the most common cause of persistent anovulation (2). PCOS consists of infertility, oligomenorrhea or amenorrhea, acne, hirsutism, and obesity (3). Ovulation occurs in 60%-80% of patients with PCOS in response to Clomiphene Citrate (CC), which is the first option to induce ovulation (4). If anovulation persists or pregnancy does not occur, other medications may be added to the regime to induce ovulation (5, 6) Insulin resistance (hyperinsulinemia) causes the reduced production of sex hormone binding globulin (SHBG) in the liver, the overproduction of ovarian and peripheral androgen, and an increase in luteinizing hormone levels that manifest as anovulation in PCOS patients (7). Targeting these metabolic disorders enhances ovulation and fertility in these women. The efficacy of treatment with insulin sensitizers, such as Metformin (Met), alone or in combination with CC is equal or superior to that of CC alone (8, 9).

Theoretically, insulin sensitizer therapy such as Met decreases hyperandrogenism and hyperinsulinemia and leads to a normal ovulatory cycle in women with PCOS (10). Some studies have reported the beneficial effects of combined Met-CC therapy in CC-resistant PCOS patients that significantly improved the ovulation rate, and even noted that Met-therapy significantly improved most outcome parameters (11, 12). Other trials, however, have shown that the addition of Met indicated ovulation rates were observed (13, 14). Also, researchers disagreed about pregnancy rates and live births in both arms. Some concluded that patients treated with CC + Met were significantly less likely to achieve pregnancy) or live birth compared with patients treated with CC and placebo or CC alone (1, 15); others reported it was higher (16, 17). Due to Met being significantly less expensive than gonadotropins, ovulation induction with Met-CC can be advocated prior to the initiation of treatment with gonadotropins. Thus the aim of this study was to evaluate the efficacy of Met added to CC in comparison to CC alone in improving ovulation. Pregnancy outcome was not a primary aim of our study.

## Materials and Methods

This prospective single-blind randomized control trial was performed on 334 infertile PCOS patients at two infertility centers affiliated with Babol University of Medical Science, Northern Iran, between 2007 and 2009. Inclusion criteria included patients between the ages of 18-35 years with duration of infertility less than five years, diagnosed with PCOS, and who were candidates for intra-uterine insemination (IUI). The diagnosis of PCOS was based on the Rotterdam criteria (at least two of the following three criteria were used); chronic anovulation and clinical or biochemical signs of hyperandrogenism, also Polycystic ovarian morphology as shown on an ultrasound scan, the presence of >12 follicles (with one ovary being sufficient for diagnosis, measuring 2-9 mm in diameter), normal values of thyroid-stimulating hormone (TSH) and prolactin, and normal renal (creatinine levels) and liver function (SGOT, SGPT) tests (18). Patients with histories of liver and kidney failure, cardiovascular disease, diabetes (based on American Diabetic Association criteria), hyperprolactinemia, thyroid disease, endometriosis, tubal or male factor, and patients with a background of Met side effects were excluded from the study. Patients were groups according to body mass index (BMI) as follows: 1. underweight (<18.5 kg/m^2^), 2. normal weight (18.5-24.9 kg/m^2^), 3. overweight (25-29.9 kg/m^2^) and 4. obese (≥ 30 kg/m^2^) to determine which BMI range responded to treatments and achieved pregnancy.

### Ovulation induction

This study was approved by the Ethics Committee of the Babol University of Medical Science. All patients were candidates for IUI and signed informed consents before entering the study. Patients were assessed by transvaginal ultrasonography (TVS) on the third day of menstruation (5 MHz probe Fokuda, Japan) to rule out ovarian cysts. Serum FSH and LH levels were also evaluated on the third cycle day. Patients were randomly allocated into two groups (167 in each group). Both groups were matched for age, duration of infertility, and BMI. We administered CC (50 mg, Iran Hormone, Tehran) in two doses/day from the third day of the menstrual cycle for up to five days for both groups. The study group also received 500 mg Met (Apotex Inc., Toronto, Canada) in three doses/day. Treatment was continued for up to three cycles. Patients were followed with TVS to document follicle growth and endometrial response. When they had at least one dominant follicle (16-22 mm) human chorionic gonadotropin (HCG, Darou Pakhsh Co.) 5000 IU was administered intramuscularly and the patients underwent IUI 36-38 hours later. Patients were recommended to perform a β-HCG test 16 days after IUI. Confirmation of pregnancy was achieved by the presence of a Yolk sac as seen on ultrasound. The number of mature follicles, pregnancy rate, and percentage of patient’s who responded in each treatment cycle (who had a dominant follicle of 16-22 mm) were evaluated.

## Results

This study included 334 PCOS patients. Clinical criteria are shown in table 1. In the present study, the minimum number of dominant follicles on the HCG injection day was one and the maximum was three. The mean dominant follicles in PCOS patients in the CC and CC + Met groups are shown in table 1.

**Table 1:** Clinical characteristic and hormonal profile in The Clomiphene (CC) group and Metformin-Clomiphene citrate (Met + CC) group


	CC (n=167)	Met+CC (n=167)

**Age (years)**	25.26±4.32	24.86±3.78
**BMI(kg/m^2^) **	25.42±3.82	26.25±4.05*
**Duration of infertility (years)**	2.67±1.33	2.36±1.43
**FSH (IU/L) **	5.6±2.8	5.4±2.4
**LH (IU/L) **	6.6±3.9	7.5±4.6
**Number of dominant follicles**	1.36±0.5	1.7±0.68*


Mean ± SD. * p≤0.05.

Reproductive clinical outcomes in the study groups are shown in table 2. All patients in the CC + Met group that had a dominant follicle were candidates for IUI at the end of the second cycle, which was significant (p<0.05; Table2). Primary infertility was noted in 264 (79%) patients, whereas 70 (21%) had secondary infertility. There were 182 (54.5%) patients that had signs of hirsutism and 173 (51.8%) with irregular menstruation. In 12 (3.6%) patients the BMI was less than normal, 130 (38.9%) were normal, 140 (41.9%) had a BMI greater than normal, and 52 (15.6%) were obese.

**Fig 1:**
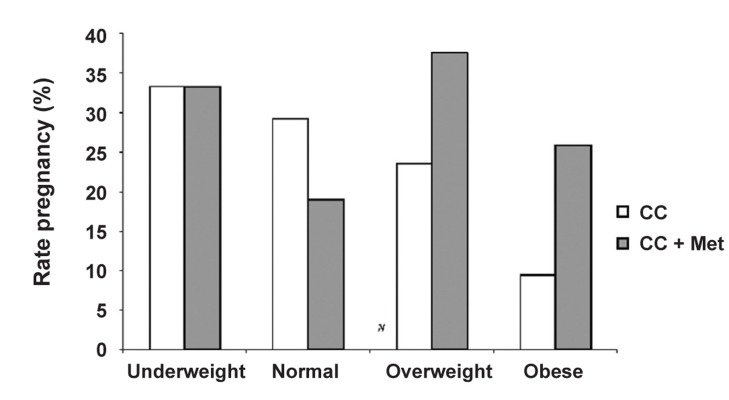
Pregnancy rate in different BMI classifications in PCOS patients in the study groups.

The pregnancy rate in different classifications of BMI in PCOS patients in the study groups showed that Met improved the rate of pregnancy in overweight and obese patients, but it was not significant (Fig 1). There was a significant difference between the mean BMI in the CC + Met group and in the CC group in pregnant women (p<0.05; Table 3). Meanwhile, 71 women (26.9%) with primary infertility and 18 women (25.7%) with secondary infertility became pregnant. There were no reports of Met-related side effects.

**Table 2:** Reproductive clinical outcomes in the study groups


	CC (n=167)			Met + CC (n=167)		
	Cycle 1	Cycle 2	Cycle 3	Cycle 1	Cycle 2	Cycle 3

**At least one dominant follicle in each cycle**	54.5% (91)	31.7% (53)	13.8%(23)	68.3% (114)*	31.7 (53)	0*
**A minimum of 2 dominant follicles (Total)**	_	34.7% (58)	_	_	57.5% (96)*	_
**Pregnancy in each treatment cycle**	58.5% (24)	24.4% (10)	17.1%(7)	64.6% (31)	35.4% (17)*	0
**Pregnancy rate (Total)**	24.6% (41)	_	_	28.7% (48)	_	_


* p≤0.05.

**Table 3:** Pregnant characteristics in the study groups


	CC (n=41)	Met+CC (n=167)

**Age (Year)**	26 ± 4.51	24.54± 3.78
**Infertility duration(Year)**	2.43 ± 1.18	2.46 ± 1.54
**BMI (Kg/m^2^)**	24.92 ± 3.57	26.76± 3.97*


Mean ± SD. * p≤0.05.

### Statistical analysis

Statistical analysis was performed by SPSS 15.0. We used the t-test and , chi-square test to calculate the results. p<0.05 was considered significant.

## Discussion

In our study, there was a significant difference between patients in the CC + Met group that had at least one mature follicle in treatment cycles and were candidates for IUI compared to patients in the CC group.

Our results agreed with studies which reported significant improvements in ovulation with Met therapy in CC-resistant women with PCOS (10, 19). Ben ayed concluded that ovulatory response to CC was increased by decreasing insulin secretion with Met (20). Kazerooni et al said "as Met seems to initiate orderly follicular growth, it may offer a reasonable therapeutic option before or after CC treatment and before starting with laparoscopic ovarian drilling (LOD) or follicular stimulating hormone (FSH)" (21). Dasari et al. (16) conducted a similar study and concluded that Met increased ovulation and pregnancy rate in CC failures. In their study, the sample size consisted of nine women who received Met for six months, while in our study the sample size was 167 women that were administered Met for three months. In the first course with CC + Met, the ovulation rate was 68% in our study and 55% in Dasari’s study, but total patients in his study ovulated during the fourth cycle, while in our study, whole patients ovulated up to the second cycle). In their study, 11% ovulated with CC100 alone while in our study the number was 31.7%. This ratio seemed identical because of the high sample size and high ovulatory by CC alone in our control group. Siebert et al. however, had conflicting results; they reported the ovulation rate achieved in women given Met + CC was similar to those only given CC (11.12% vs. 11.14%). In their study, group A (52 women) received pre-treatment with Met for six weeks before adding CC and group B (55 women) received CC alone. In both groups, CC was increased to a maximum of 150 mg if no response was achieved after four cycles (2). The main difference between their study and ours was the pre-treatment of Met Sturrock et al and Moll et al failed to find an increase in ovulation rate of combined therapy compared with CC alone. (12, 22) According to Moll, the effects of Met on ovulation might not be sufficiently strong enough to improve on the already high ovulation rates with CC in these women (22). As insulin resistance did not improve substantially, this theory seems reasonable. It is possible that women who ovulate on Met monotherapy would also ovulate on CC monotherapy, thus explaining the absence of an added effect). Zain et al. study has demonstrated that the addition of Met to CC does not significantly increase ovulation, pregnancy, or live birth rate, although there was a slight increase in the three parameters when compared to CC. He concluded that CC should be the first-line treatment for ovulation induction in anovulatory patients with PCOS. In their study, if there was an absence of ovulation, the CC dose was increased stepwise to a maximum of 200 mg. The success rate of CC in their study was possibly due to the enhanced dose of CC. Also in their study, if there was evidence of ovulation but the patient did not become pregnant, the same dosage was continued for a maximum of six cycles, although, high and long-term doses of CC may cause some complications (13).

In our study, there was no significant difference in the rate of pregnancy (24.6% vs. 28.7%, p>0.05) in the CC + Met group compared to the CC group. In the Palomba et al. study the pregnancy rate was significantly higher in the Met group than in the CC group (15.1 vs. 7.2%) (1). Heard et al. (23), Malkawi and Qublan showed similar results (24). A high significant pregnancy result has been limited to a few studies (14, 25). Other studies have reported less or equal pregnancy rate or live birth by adding Met to CC which are compatible with our study. (16, 17) These results may be due to the fact that pregnancy is very complex and depends on multiple factors. However, Basirat et al have reported in a study that the number of dominant follicles do not correlate with the outcome of pregnancy (26).

Our study has shown that the mean BMI in the CC + Met group was higher than in the CC group in pregnant women, which seemed to improve the pregnancy rate in overweight and obese patients. Mol et al. have reported the same results in obese patients (22). It seems that Met is more effective in women who have a higher BMI. Possibly Met assists these women to become pregnant no waiting to lose weight. It’s notable that weight loss in PCOs women is associated with taking a lot of time and sometimes seems to be out of patients' tolerance. Although, Ben Ayed and colleagues have shown that no significant difference in the treatment effects were found for groups based on BMI (20). Also, Ng et al. studied twenty infertile PCOS women remained anovulatory on CC and randomized them to receive placebo or metformin 500 mg. Clomiphene was then added for one cycle to those women who did not ovulate after taking placebo or metformin alone. There was no improvement in the ovulation rate despite a significant reduction of body mass index (27). While, Baillargeon concluded that in obese women with PCOS, Met possibly improved the action of insulin in part by improving insulin-mediated release (28). In the Qublan study, Met monotherapy was effective in CC-resistant women with morbid obesity and primary infertility. In other words, there is a superiority of the ovulation rate occurring in PCOS women with high BMI; hence we suggested it should be considered as the first-line treatment in these patients (29).

## Conclusion

The findings of the present study suggest that while our approach significantly increases follicular growth in these women without a higher pregnancy rate by adding Met, it may offer a reasonable therapeutic option when combined with CC. Another study with larger numbers of participants should be undertaken in order to clarify the impact of Met on pregnancy rates and to obtain more attention before clinical recommendations. A well-designed randomized controlled trial needs to clarify the value of a long and short course of Met treatment on pregnancy outcomes of PCOS patients. However a limitation to our study was not using Met as a pretreatment drug in order to compare ovulation and pregnancy rates.
